# Supplementary material for: VARX Granger analysis: Models for neuroscience, physiology, sociology and econometrics
Source: PLoS One. 2025 Jan 9;20(1):e0313875. doi: 10.1371/journal.pone.0313875 (PMC11717226; doi:10.1371/journal.pone.0313875)
Supplement: S1 File — (PDF) [file pone.0313875.s001.pdf]

S1: Validation of p-values with L2 regularization

As T increases the need for regularization decreases, so we scale the regularization factor as $\gamma = \lambda/\sqrt{df}$, where λ is a choice of regulation in the order of 1. This particular scaling was established empirically to accomplish two things. One, if γ is kept large for large T, then the asymptotic approximation used to compute the de-biased deviance is no longer correct and p-values are miss-estimated. Second, the specific scaling with the square root is an empirical finding that places the optimal γ at similar values of λ in simulation with different signal lengths T. In Fig 1 we simulated a simple model for varying λ and T. In this example we find, as expected, that training error increases with regularization (Fig 1a), but test error decreases with increasing regularization (Fig 1b). This effect becomes clear with larger noise (note that the explained variance here is relatively small) and with increasing correlation in input (here random normal inputs were drawn independently). The second observation is that with increasing sample size T, test error drops, and regularization becomes less important. Finally, we see that the bias correction provides conservative control of the false discovery rate (Fig 1c). The expected value in this example is 0.05 and at small T we are well below that. Indeed, for small T we seem to be over-correcting, which limits the power to detect a true effect Fig 1d).

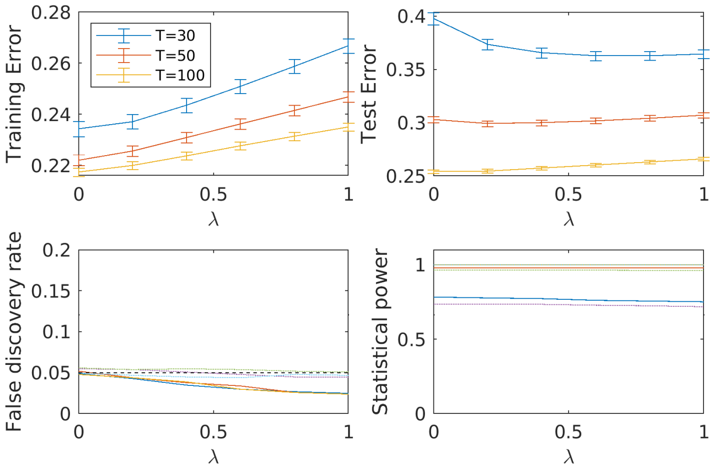

Fig 1. Effect of L2 regularization on small toy examples $d_y = 2, d_x = 2$. We ran the model and fit the data computing analysis of p-values in 1000 simulations with normal distributed error (zero mean, std of 2) and inputs x (zero mean, std of 1). (a) the training error is the relative error, i.e. $\sigma_e^2/std(y)^2$ computed on the same data that the model was fit on (length T samples). Error bars indicate SEM over the 1000 simulations. (b) test error is the same relative error but computed on newly simulated data with the estimated model. (c) False discovery is the fraction of times where the null path is $p < 0.05$. Solid lines are for false discovery in $\mathbf{A}$, and dotted lines are for false discovery in $\mathbf{B}$ in this simulation. (d) Statistical power is the fraction of times that the $p > 0.05$ for the path that has non-zero coefficients. The specific models tested here had effects of $y_1 \rightarrow y_2$ and $x_2 \rightarrow y_2$ set to zero and otherwise $n_a = 2, n_b = 10$ coefficients with non-zero values.
