# Supplementary material for: VARX Granger analysis: Models for neuroscience, physiology, sociology and econometrics
Source: PLoS One. 2025 Jan 9;20(1):e0313875. doi: 10.1371/journal.pone.0313875 (PMC11717226; doi:10.1371/journal.pone.0313875)
Supplement: S2 File — (PDF) [file pone.0313875.s002.pdf]

S2: Validation of p-values when using Basis Functions

For large T there is minimal regularization and regularization can not be used to "smooth" the estimated filters. For that, we developed the basis functions decomposition using smooth basis functions. A second motivation was also to make the number of parameters of **A** and **B** filters comparable, as we noticed empirically that an imbalance results in quite skewed p-values. We simulated examples with very different filter lengths $n_a = 2, n_b = 60, n = 6$ (Fig 1). We find that introducing basis functions improves the estimates of false discovery rate while providing smooth filter estimates. We noticed empirically that these basis functions, for slow signals, made the signals significantly larger in power. As a result, conventional ridge regression regularized different signals differently, resulting in a miss-estimation of values. This motivated the adoption of Tikhonov regularization so that all variables are regularized by the same amount.

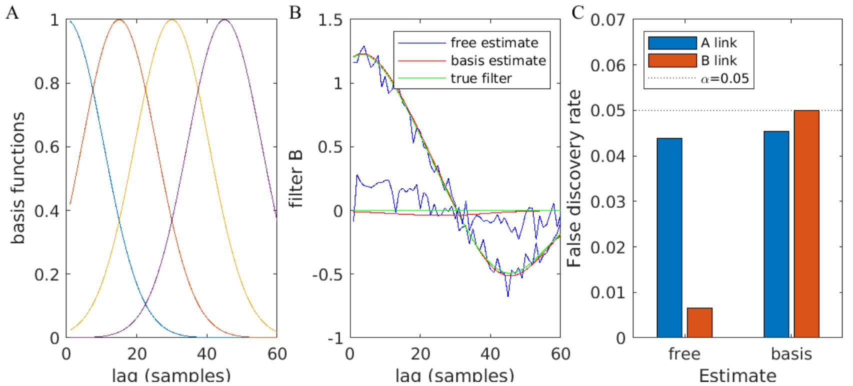

Fig 1. Estimate MA filter coefficients **B** with and without basis functions (basis vs free, respectively). (A) The Gaussian basis functions are used here, both for the generation of the data and for estimation. (B) Corresponding filter estimates and true filters used during data generation. Here $d_y = 2$ and $d_x = 1$, with $y_1 \rightarrow y_2$ and $x \rightarrow y_2$ channels set to zero. Data was simulated with $T = 300$ and $n_a = 3$, $n_b = 60$, $n_b ar = 4$. The total number of free parameters was $4 * n_a + 2 * n_b = 132$ (free) and $4 * na + 2 * \underline{n} = 20$ (basis). No regularization was used. (C) False discovery rate for AR and MA channels with and without basis functions. False discovery (bars) indicates how many times in 5000 random simulations these missing links reported a $p < 0.05$, therefore we expect the discovery rate to be 0.05 (dotted line).
