# Supplementary material for: VARX Granger analysis: Models for neuroscience, physiology, sociology and econometrics
Source: PLoS One. 2025 Jan 9;20(1):e0313875. doi: 10.1371/journal.pone.0313875 (PMC11717226; doi:10.1371/journal.pone.0313875)
Supplement: S3 File — (PDF) [file pone.0313875.s003.pdf]

S3: De-biased Deviance under L2 Regularization

To our knowledge, the correction term for the deviance in the case of L2 regularization of a linear estimator is not yet available in the literature. We therefore detail here its derivation.

Consider the linear model for $n_y = 1$:

$$\mathbf{y} = \mathbf{X} \cdot \mathbf{h} + \mathbf{e}, \tag{1}$$

for simplicity of exposition. The case of $n_y > 1$ is a natural generalization, as we explain below. A ridge regressor for $\mathbf{h}$ is obtained by:

$$\hat{\mathbf{h}} := \underset{\mathbf{h}}{\operatorname{argmin}} \quad \|\mathbf{y} - \mathbf{X} \cdot \mathbf{h}\|^2 + \gamma \|\mathbf{h}\|^2, \tag{2}$$

where γ is the ridge parameter. To perform de-biasing, stationarity conditions for $\hat{\mathbf{h}}$ give:

$$-\mathbf{X}^\top (\mathbf{y} - \mathbf{X}\hat{\mathbf{h}}) + \lambda \hat{\mathbf{h}} = \mathbf{0}. \tag{3}$$

Following the arguments of Eqs. (3)–(5) in [1], the following estimator is the de-biased estimator:

$$\hat{\mathbf{h}}^{de-biased} = \hat{\mathbf{h}} + (\mathbf{X}^\top \mathbf{X})^{-1} \mathbf{X}^\top \left(\mathbf{y} - \mathbf{X}\hat{\mathbf{h}} \right) = \hat{\mathbf{h}} + \mathbf{R}_{xx}^{-1} \mathbf{r}_{xe}. \tag{4}$$

Now, consider the GC inference setting where we have full and reduced models given by:

$$\text{Full model:} \quad \mathbf{y} = \mathbf{X}^f \mathbf{h}^f + \mathbf{e}^f, \quad \text{Reduced model:} \quad \mathbf{y} = \mathbf{X}^r \mathbf{h}^r + \mathbf{e}^r. \tag{5}$$

The log-likelihoods of the full and reduced models are given by:

$$\ell^f := -\frac{T}{2} \ln(2\pi) - \frac{T}{2} \ln \sigma_f^2 - \frac{1}{2\sigma_f^2} \left\| \mathbf{y} - \mathbf{X}^f \mathbf{h}^f \right\|^2, \tag{6}$$

$$\ell^r := -\frac{T}{2} \ln(2\pi) - \frac{T}{2} \ln \sigma_r^2 - \frac{1}{2\sigma_r^2} \left\| \mathbf{y} - \mathbf{X}^r \mathbf{h}^r \right\|^2, \tag{7}$$

and the deviance difference is given by $\mathscr{D} := 2\big(\hat{\ell}^f - \hat{\ell}^r\big)$, where $\hat{\ell}^f$ and $\hat{\ell}^r$ are, respectively, the full and reduced log-likelihoods evaluated at the ridge estimates of full and reduced parameters:

$$\hat{\mathbf{h}}^f := \underset{\mathbf{h}}{\operatorname{argmin}} \quad \|\mathbf{y} - \mathbf{X}^f \mathbf{h}\|^2 + \lambda \|\mathbf{h}\|^2, \tag{8}$$

$$\hat{\mathbf{h}}^r := \underset{\mathbf{h}}{\operatorname{argmin}} \quad \|\mathbf{y} - \mathbf{X}^r \mathbf{h}\|^2 + \lambda \|\mathbf{h}\|^2. \tag{9}$$

If the log-likelihoods were evaluated at the ML estimators, then $\mathscr{D}$ would be asymptotically chi-square/non-central chi-square, allowing precise statistical tests. But the ridge estimator is different from the ML estimator. So, we need to de-bias the deviance difference.

Following Eq. (20) and Eqs. (B.2)–(B.6) in [2], the de-biased deviance difference is given by:

$$\mathscr{D}^{de-biased} = 2\big(\hat{\ell}^f - \hat{\ell}^r\big) - B_r + B_f, \tag{10}$$

where the bias function $B(\cdot)$ is defined as:

$$b_f := \frac{1}{2\hat{\sigma}_f^2} \big(\mathbf{y} - \mathbf{X}^f \hat{\mathbf{h}}^f\big)^\top \mathbf{X}^f \left(\mathbf{X}^{f\top} \mathbf{X}^f\right)^{-1} \mathbf{X}^{f\top} \big(\mathbf{y} - \mathbf{X}^f \hat{\mathbf{h}}^f\big) = \frac{1}{2r_{ee}^f} \mathbf{r}_{xe}^{f\top} \mathbf{R}_{xx}^{f\,-1} \mathbf{r}_{xe}^f, \tag{11}$$

$$b_r := \frac{1}{2\hat{\sigma}_r^2} \big(\mathbf{y} - \mathbf{X}^r \hat{\mathbf{h}}^r\big)^\top \mathbf{X}^r \left(\mathbf{X}^{r\top} \mathbf{X}^r\right)^{-1} \mathbf{X}^{r\top} \big(\mathbf{y} - \mathbf{X}^r \hat{\mathbf{h}}^r\big) = \frac{1}{2r_{ee}^r} \mathbf{r}_{xe}^{r\top} \mathbf{R}_{xx}^{r\,-1} \mathbf{r}_{xe}^r. \tag{12}$$

Simplifying the deviance difference $\mathscr{D}$ for the linear model, we can express $\mathscr{D}^{de-biased}$ as:

$$\mathscr{D}^{de-biased} = T \ln \frac{\hat{\sigma}_r^2}{\hat{\sigma}_f^2} - b_r + b_f, \tag{13}$$

where

$$\hat{\sigma}_f^2 := \frac{1}{T} \left\| \mathbf{y} - \mathbf{X}^f \hat{\mathbf{h}}^f \right\|^2 = r_{ee}^f, \qquad \hat{\sigma}_r^2 := \frac{1}{T} \left\| \mathbf{y} - \mathbf{X}^r \hat{\mathbf{h}}^r \right\|^2 = r_{ee}^r, \tag{14}$$

are the full and reduced prediction variances. For $n_y > 1$, given that each channel of $\mathbf{Y}$ is treated separately, the full and reduced bias vectors are given by:

$$\mathbf{b}_f = \frac{1}{2}\mathbf{diag}(\mathbf{R}_{xe}^{f\top} \cdot \mathbf{R}_{xx}^{f\ -1} \cdot \mathbf{R}_{xe}^f)/\mathbf{diag}(\mathbf{R}_{ee}^f), \mathbf{b}_r = \frac{1}{2}\mathbf{diag}(\mathbf{R}_{xe}^{r\top} \cdot \mathbf{R}_{xx}^{r\ -1} \cdot \mathbf{R}_{xe}^r)/\mathbf{diag}(\mathbf{R}_{ee}^r), \tag{15}$$

and the de-biased deviance vector is given by:

$$\mathscr{D}^{de-biased} = T \log\left(\hat{\boldsymbol{\sigma}}_r^2/\hat{\boldsymbol{\sigma}}_f^2\right) - \mathbf{b}_r + \mathbf{b}_f. \tag{16}$$

Under the null hypothesis, the de-biased deviance difference can be shown to be asymptotic, as $T \to \infty$, chi-square distributed with n degrees of freedom, where n is the number of parameters removed in the reduced model and T is the number of observations (i.e., column dimension of $\mathbf{Y}$) [3]. This asymptotic property requires that the ridge estimator is consistent. To guarantee this, γ must grow slower than $\mathcal{O}(T)$. In practice, fixing γ using cross-validation typically meets this condition.

References

1. Geer Svd, Bühlmann P, Ritov Y, Dezeure R. On asymptotically optimal confidence regions and tests for high-dimensional models. The Annals of Statistics. 2014;42(3):1166–1202. doi:10.1214/14-AOS1221.

2. Soleimani B, Das P, Dushyanthi Karunathilake IM, Kuchinsky SE, Simon JZ, Babadi B. NLGC: Network localized Granger causality with application to MEG directional functional connectivity analysis. Neuroimage. 2022;260(119496):119496.

3. Davidson RR, Lever WE. The Limiting Distribution of the Likelihood Ratio Statistic under a Class of Local Alternatives. Sankhyā Indian J Stat Ser. 1961;32:209–224.
