# Supplementary material for: VARX Granger analysis: Models for neuroscience, physiology, sociology and econometrics
Source: PLoS One. 2025 Jan 9;20(1):e0313875. doi: 10.1371/journal.pone.0313875 (PMC11717226; doi:10.1371/journal.pone.0313875)
Supplement: Attachment — Submitted filename: PlosOne response.pdf [file pone.0313875.s004.pdf]

ACADEMIC EDITOR:

> **Thank you for your editorial comments and suggestions. We have now addressed all these points in response to the comments of Reviewers 1 and 2, as detailed below. Despite the request for "minor revisions" the reviewer comments prompted us to add significant new text to further improve the presentation and rigor of our manuscript, which is marked in blue.**

Comments to the Author

Reviewer 1:

General Comments:
This article studies the disentangling delayed external effects from internal dynamics. The authors use a Vector Autoregressive model with eXogenous input (VARX) by providing fundamental equations, user-friendly code, and demonstrations using simulated and real-world data from neuroscience, physiology, sociology, and economics. The authors reached the conclusions that model avoids spurious correlation by factoring out external influences from internal dynamics, leading to more parsimonious explanations of the systems with methods for enhancing model efficiency, such as L2 regularization for limited data and basis functions to cope with extended delays

Overview:
The paper is good written and the empirical work does appear to be carefully and correctly done. The research question is somehow good and it does make a sufficient new contribution to the literature to be suitable for the PLOS ONE ONLY after MINOR revisons.
In fact, the use of VARX Granger Analysis: Modeling, Inference, and Applications is quite new in the literature.
The contribution of the paper is the use of a Vector Autoregressive model with eXogenous input (VARX) by providing fundamental equations, user-friendly code, and demonstrations using simulated and real-world data from neuroscience, physiology, sociology, and economics.
The paper is neutral interesting; and in my view, it needs to be MINOR improved to reach the standard required for publication in this journal.

> **Thank you for your positive comments and for recommending minor revisions.**

Specific Comments:

1. Title: introduce the subjects for application

> **We have changed the title to the following to make it more specific:**
>
> VARX Granger analysis: Models for neuroscience, physiology, sociology and econometrics

2. Abstract: somehow large, try to reduce and introduce with the present results from the article

The following are specific results that are now mentioned in the abstract:

Our examples illustrate how the model avoids spurious correlation by factoring out external influences from internal dynamics, leading to more parsimonious explanations of these systems. For instance, in neural recordings we find that prolonged response of the brain can be explained as a short exogenous effect, followed by prolonged internal recurrent activity. In recordings of human physiology we find that the model recovers established effects such as eye movements affecting pupil size and a bidirectional interaction of respiration and heart rate.

Please note that this is a paper on a new method and so the emphasis in the Abstract is on the new methodological contributions, not the results on specific applications, which are presented only for demonstration purposes, are are not intended as in-depth analysis in a particular field.

3. Introduction: NOVELTY + results (better explanation); enlarge at least 1.5-2 pages

We have clarified in the last section of the Introduction to highlight the the novel aspects of our work:

The following are the novel contributions of this work. First, we treat exogenous variables in their own right by applying the Granger formalism to VARX models. By doing this, we are capturing the lagged effects of the exogenous input, and separating that from the internal dynamics. To enable this, we present the basic equations required to estimate VARX model parameters, effect size, and statistical significance, based on Deviance. Then, we demonstrate the validity of this approach using simulated data. We further explore methods, such as L2 regularization and the usage of basis functions in parametric modeling, to handle high-dimensional datasets and obtain longer prediction filters. These methods effectively reduce the number of parameters. We present in the Appendix the first derivation of de-biased estimates of the test statistic for L2 regularization. We will also illustrate instances where interpreting the Granger formalism as ``causal'' can be misleading, such as in cases involving missing variables or colliders. Subsequently, we showcase examples that apply this formalism to neural signals, highlighting the key differences between VARX models and "temporal response functions" [35] commonly used in neuroscience. We will also present examples using physiological, sociological, and economic data, treated here for the first time with the VARX formalism. Finally, we conclude with a discussion on the specifics of code implementation and some caveats regarding the interpretation of model results.

4. Literature review: at least 2 pages

We have now expanded the literature review in the introduction, by giving an overview of the various methods for parametric and nonparametric time series analysis and their relevance to our contributions.

In practice, the optimal linear filters are estimated using ordinary least squares. Then, the logarithmic ratio of the two prediction error variances, in presence and absence of x(t) and its past, is taken as the test statistic and its statistical significance is assessed based on the corresponding asymptotic distributions [5–7]. While this procedure is relatively simple to perform, it faces a key challenge: in order to obtain reliable parameter estimates via least squares, a typically long observation window is required. In datasets with short duration, the foregoing models typically over-fit the observed data, resulting in unreliable parameter estimates [8, 9].

This challenge has been addressed in the context of regularized estimation [10–19], in which the least squares objective function is augmented with a penalty term that enforces additional restrictions on the parameters, such as smoothness, sparsity, and low-rank structures. While some of these sophisticated regularization schemes, such as the L1-regularization or the LASSO [20, 21], smoothly clipped absolute deviation [22, 23], Elastic-Net [24], and their variants, and have particularly proven useful in VAR estimation [11–13, 17, 18], regularization with the ℓ2-norm [10, 25], also known as “ridge regression”, is arguably the most widely-used in practice, due to the simplicity of the the resulting parameter estimator.

It is worth noting that in parallel to the aforementioned parametric models, some existing nonparametric methods bypass VAR estimation by instead employing techniques such as spectral matrix factorization [26] or multivariate embedding [27].In terms of statistical testing of the effects, partial correlation-based nonparametric methods that employ conditional independence tests have also been suggested [28–30], which require no time-series modeling assumptions.

5. Methodology: why the authors use only these indicators into the model? Present some solid theoretical explanations for these indicators. Enlarge at least 2 pages.

Variables used in individual applications are described in the Results section (Sections 3.3-3.6). Rather than expanding on this in the Methods section, which focuses on math, we are expanding on this in the Results. But first, we added the following at the start of the Results, in order to set the appropriate expectations for the readers:

We will start with a few examples on simulated data to demonstrate the validity of the approach (Section 3.1 and 3.2). We then analyze real-world data from neuroscience, physiology, sociology and macroeconomics (Section 3.3-3.6). These examples are not meant as in-depth analyses in these diverse disciplines, but as demonstrations of how

the VARX Granger analysis can be used in principle. More detailed validation and extensions are left for an Appendix (Section 7).

Below we reproduce existing description and motivation of the variable used, and include additional clarifications (in blue):

In Section 3.3:

We use the neural data from [38], and use the same preprocessing to extract high-frequency broadband activity (BHA, 70-150Hz, downsampled to 60Hz), often considered a marker of local neuronal firing. The exogenous "input" is a pulse train indicating fixation starts (moments of new visual input). Of course, multiple other features of the video stimulus could have been used as "input". Previous research with natural speech and movies has used features such as sound volume, visual motion, or specific content of the video and sound [33,34]. By focusing on fixation onset, we intend to extract the neural activity associated with the initial visual processing, known as core vision [41].

In Section 3.4:

Human physiology is a dynamic system with multiple dependent signals. In previous work we reported correlations between respiration, heart rate, pupil response, and brain activity [42]. We were motivated to identify in these signals interactions between body and mind. Pupil size and heart rate were measured in the experiment as they are well established metrics of physiological arousal. Respiration was measured because it is well known to affect heart rate, and eye movements were analyzed because of their association with arousal [43].

In Section 3.5:

Here, we present an analysis from the field of sociology. We examine the history of workers' union membership and its relationship to strikes (Fig. 6A). We hypothesize that strikes increase union membership in subsequent years. The variables here were specifically selected to test this hypothesis, largely following [46]. We assume the unemployment rate is unaffected by union variables, so it is modeled as an exogenous input. In contrast, the number of unionized workers, the number of workers on strike, and the number of strikes can all potentially influence each other.

In Section 3.6:

As a final example, we demonstrate the model on a dataset from the U.S. Federal Reserve encompassing fiscal, monetary, and labor factors, spanning quarters from 1959 to 2009. These data were selected because of their availability in the Econometrics Toolbox in Matlab. Here we have converted all gross numbers into annual percentage rates. This removes the exponential growth resulting from predominantly positive rates (Fig.~\ref{US-Economy}A), which leads to trivial correlations and non-stationarity

(sometimes referred to as unit-root signals). To determine the effect of the government on the economic variables, we examined the impact of government spending (GCE) and federal funds rate (FEDFUND). Government spending itself is a function of economic conditions, such as unemployment benefits, which are automatically linked to unemployment, while a rise in GDP increases tax revenue, which typically leads to increased government spending. Nevertheless, by treating GCE and FEDFUND as exogenous variables, we are asking what effects these government policies have on the economy, if they were controlled independently.

Incidentally, we decided to invite an additional author who contributed to Section 3.5 by formulating the hypothesis, providing literature and compiling the data from various sources. Previously she had only been mentioned in the Acknowledgments. Now she is listed as co-author: Vera E Parra.

6. Section 4: The analysis is very small and simplistic; enlarge this part of the article to at least 3 pages

Perhaps the formatting of Section 4, with many short subsections, was confusing and gave the impression that there is only a short analysis. We have reformatted this section to make clear that the following topics are all part of Section 4, which indeed now cover 3 pages:

Novel contributions, Related toolboxes, Equation error versus output error models, Comparison of VARX, MA, OE, and VAR models, Sensitivity to parameters, Caveats to Causality, Non-stationarity, Stability, 1/f Spectrum

7. Discussions: at least 2 pages; separate from the conclusions

The Discussion is now 3 pages. We also included the following Conclusions, separate from the Discussion.

5. Conclusion

The predominant approach to modeling the effect of exogenous variables onto a dynamical system is to simply treat them as input and output of a vectorial MA filter (known as "temporal response function" in neuroscience, or simply "impulse response" in the linear systems literature). Unlike the VAR model, this is not commonly examined in the Granger formalism. Although Granger and Geweke both incorporate exogenous variables into the analysis formalism [Geweke 1984], the connection to the VARX model has not yet been widely recognized. We hope to have bridged this gap. While not incorrect, the simple MA approach fails to factor out the portion of the total system response that is due to the internal dynamic, and separates that from the external drive. In contrast, when relying only on VAR models, one fails to exploit the prior knowledge

that some variables are independent of the internal dynamic. In summary, different models vary in their assumptions about how to break down the system's overall response. When estimating with the VARX model, we manage to uniquely factor the overall response into external drive versus internal dynamics.

8. The article is extremely detailed and concentrated on results part; modify and transform into an academic one

To minimize less relevant details we have moved the more detailed analyses and derivations to an Appendix.

We hope that our revisions in response to the preceding comments address this point as well. We note that the norms of academic writing differ significantly between fields. As this is a multi-disciplinary paper, it is not feasible to abide by the conventions of any particular research domain. We have therefore chosen PLOS One which is a general journal where the structure of a paper can be more flexible. Our intention was to write the paper so that there is something for everyone, so it can be of use in many disciplines. It was not the intention to go in-depth in any one of the application areas.

Reviewer 2

General considerations:
The idea of the article is good, and the construction of the article is sometimes very mathematical and not easy to read. The authors MUST improve the literature, methodology, explanations, discussions, and change the article accordingly. The authors MUST rearrange the methodology part, enlarge the analysis, modify into a readable article.
I ONLY recommend this article be published in PLOS ONE after MINOR revisions (literature, methodology, analysis and the discussion).

Thank you for your positive assessment of our submission. We hope that the edits above and below address these points.

Reviewer #2: This study presents a novel approach for capturing temporal dynamics within a dynamic system. Specifically, the VARX model decomposes the system response into auto-regressive (AR) and moving average (MA) components, offering potential utility in the field of neuroscience.

While the authors provide detailed equations for the VARX model, the exposition on separating the AR and MA components is lacking in specificity. It is recommended that the authors elaborate on how the AR and MA components in the VARX model relate to mTRF analysis and frequency analysis in the neuroscience domain.

We have now added the following clarification in the section on the total system response:

In this view, what we are proposing is to model the total system response as a combination of a Moving Average (MA) filtering **B** and Auto-Regressive (AR) filtering $(\mathbf{I} - \mathbf{A})^{-1}$ ... The alternative is to model the total system response as a single MA filter. This is the approach taken in mTRF [35] and Neuro-Current Response Function (NCRF) [36] frameworks. We will show (in Fig. 4) that the total system response estimated either as VARX or MA models are practically the same. However, as we see in Eq. (4), the VARX model factors the total response into an exogenous and an endogenous dynamic, with parameters **B** and **A**, respectively.

The frequency analysis will be addressed in response to the next question.

Furthermore, a discussion on various mTRF toolboxes in comparison to the VARX model is warranted.

We have now expanded the section on related toolboxes, adding in particular a discussion of mTRF and related toolboxes:

Related toolboxes: There are several software tools to estimate VARX models. We are not aware of one that provides the Granger-Geweke test for significance. The MVGC toolbox [18] in Matlab supports control variables, but does not report results on exogenous variables. It therefore is mostly a tool to estimate VAR models only. The MVGC toolbox identifies parameters **A** in both time and frequency domain. To our knowledge, all implementations of VARX models identify parameters A and B only in the time domain, including our own. Implementations of VARX models in Matlab's Econometrics Toolbox and in SAS software for instance, make significance statements for individual delays but do not allow for the exogenous variable, i.e. nb = 1. Other toolboxes written in Matlab/python such as mTRF [35], NCRF [36], or Unfold [51] only identify parameters **B** of MA models, i.e. they ignore endogenous effects. To our knowledge, this is the first implementation of a model that estimates both **A** and **B** with time delays in each. Therefore we estimate both endogenous and exogenous delayed effects and emphasize computational efficiency to handle large datasets, with comparatively long filters **B**. The tool is available for Matlab, Python and R.

Lastly, the discussion on L2 regularization should be contextualized within the framework of regularization parameters in mTRF analysis.

We have now added the following discussion on L2 regularization:

L2 regularization: In this study, we proposed utilizing L2 regularization due to its compatibility with the closed-form solution of linear least squares problems. This approach enables efficient computations for large models. It is often used when estimating MA models (e.g., mTRF toolbox) as long delays add a large number of

parameters. However, all regularization methods introduce a bias in the model estimates. This introduced the need for a bias correction in parametric estimates of statistical significance [52]. Corrections are available for L1 but not for L2 regularization [52,60,61], which we presented here for the first time. L1 regularization has the advantage that it results in sparse parameterizations, and has been used in the context of VAR models [31,52,61] However, it is computationally more demanding. In contrast, L2 regularization allowed us to implement fast computations of statistical significance for each channel in **A** and **B**. These new bias correction formulas should also enable fast computation of statistical significance in MA models, which so far have not been available in existing toolboxes, e.g. mTRF or Unfold [35,44].

While revising your submission, please upload your figure files to the Preflight Analysis and Conversion Engine (PACE) digital diagnostic tool, https://pacev2.apexcovantage.com/. PACE helps ensure that figures meet PLOS requirements. To use PACE, you must first register as a user. Registration is free. Then, login and navigate to the UPLOAD tab, where you will find detailed instructions on how to use the tool. If you encounter any issues or have any questions when using PACE, please email PLOS at figures@plos.org. Please note that Supporting Information files do not need this step.

Please include the following items when submitting your revised manuscript:

- A rebuttal letter that responds to each point raised by the academic editor and reviewer(s). You should upload this letter as a separate file labeled 'Response to Reviewers'.
- A marked-up copy of your manuscript that highlights changes made to the original version. You should upload this as a separate file labeled 'Revised Manuscript with Track Changes'.
- An unmarked version of your revised paper without tracked changes. You should upload this as a separate file labeled 'Manuscript'.

  We are submitting all 3 documents.

1. When submitting your revision, we need you to address these additional requirements.

Please ensure that your manuscript meets PLOS ONE's style requirements, including those for file naming. The PLOS ONE style templates can be found at https://journals.plos.org/plosone/s/file?id=wjVg/PLOSOne_formatting_sample_main_body.pdf and

https://journals.plos.org/plosone/s/file?id=ba62/PLOSOne_formatting_sample_title_authors_affiliations.pdf

We have read and adjusted to these formatting guidelines. We are providing all images in PNG (original) and TIFF (as requested).

2. Please update your submission to use the PLOS LaTeX template. The template and more information on our requirements for LaTeX submissions can be found at http://journals.plos.org/plosone/s/latex.

We are now using the PLOS One Latex template.

3. Please note that PLOS ONE has specific guidelines on code sharing for submissions in which author-generated code underpins the findings in the manuscript. In these cases, we expect all author-generated code to be made available without restrictions upon publication of the work. Please review our guidelines at https://journals.plos.org/plosone/s/materials-and-software-sharing#loc-sharing-code and ensure that your code is shared in a way that follows best practice and facilitates reproducibility and reuse.

We have read the guidelines and confirm that our shared code is in compliance.

4. We note that the grant information you provided in the 'Funding Information' and 'Financial Disclosure' sections do not match.

When you resubmit, please ensure that you provide the correct grant numbers for the awards you received for your study in the 'Funding Information' section.

5. Thank you for stating the following financial disclosure:
[LCP, ASO, JM, National Institutes of Health, NIMH P50 MH109429
LCP, JM, National Science Foundation, DRL-2201835].
Please state what role the funders took in the study. If the funders had no role, please state: ""The funders had no role in study design, data collection and analysis, decision to publish, or preparation of the manuscript.""
If this statement is not correct you must amend it as needed.
Please include this amended Role of Funder statement in your cover letter; we will change the online submission form on your behalf.

This Statement is correct. There is nothing to change.

6. Please review your reference list to ensure that it is complete and correct. If you have cited papers that have been retracted, please include the rationale for doing so in the manuscript text, or remove these references and replace them with relevant current references. Any changes to the reference list should be mentioned in the rebuttal letter that accompanies your revised manuscript. If you need to cite a retracted article, indicate the article's retracted status in the References list and also include a citation and full reference for the retraction notice.

We have reviewed the references and found no errors, and are not aware of any retractions.
